# Genetic polymorphisms in DNA repair gene XRCC1 and the risk of diabetic polyneuropathy

**DOI:** 10.1038/s41598-026-35213-1

**Published:** 2026-02-03

**Authors:** Noha A. Hashim, Hatim A. El-Baz, Zahraa I. Abo Afya, Hagar F. Gouda, Noha M. Bakr

**Affiliations:** 1 Neurology Department, Faculty of Medicine, Zagazig University, Zagazig, Egypt; 2 Biochemistry Department, Biotechnology Research Institute, National Research Centre, Dokki, Giza, Egypt; 3 Department of Clinical and Chemical Pathology, Institute of Medical Research and Clinical Studies, National Research Centre, Cairo, Egypt; 4 Animal Wealth Development Department (Biostatistics Subdivision) Faculty of Veterinary Medicine, Zagazig University, Zagazig, Egypt; 5 High Throughput Molecular and Genetic Laboratory, Center of Excellence for Advanced Sciences, National Research Centre, Dokki, Giza, Egypt

**Keywords:** Diabetic polyneuropathy, XRCC1 Arg399GLN, XRCC1 Arg194Trp, Single nucleotide polymorphism, Machine learning algorithms, Genetics, Neuroscience

## Abstract

This study aimed to investigate the association between XRCC1 Arg399Gln and Arg194Trp single nucleotide polymorphisms (SNPs) and the risk and severity of polyneuropathy (DPN) in patients with type 2 diabetes mellitus (T2DM). The genotyping of SNPs was achieved in 732 contributors, including diabetic subjects with and without polyneuropathy and controls, using polymerase chain reaction-restriction fragment length polymorphism (PCR-RFLP). In addition, by using advanced statistical techniques, including machine learning methodologies, to analyze the data.The results indicated a significant link between both SNPs and DPN risk under both codominant and dominant models, respectively, with the A and T alleles as risk variants. Haplotype analysis further established the A-T haplotype as a prominent risk factor. The disease severity was associated with the 399 A/A and combined (G/A + A/A) genotypes, as well as the 194 C/T and combined (C/T + T/T) genotypes. In advanced DPN stages, random Forest (RF) highlighted both XRCC1 SNPs, and disease duration as the top three contributing factors. SHAP analysis corroborated the 194 C/T genotype of and the 399 A/A genotype were strongly linked to severe disease manifestations, particularly when coexisting with prolonged illness duration, advanced age, elevated HDL, and reduced LDL levels. Our findings substantiate the association of XRCC1 Arg399Gln and Arg194Trp SNPs with both susceptibility to and progression of DPN in T2DM patients. The integration of machine learning methodologies augments clinical decision-making by refining diagnostic precision and facilitating personalized treatment strategies.

**Supplementary Information:**

The online version contains supplementary material available at 10.1038/s41598-026-35213-1.

## Introduction

 Diabetic peripheral neuropathy (DPN) represents a significant microvascular complication of T2DM^1^, in which vascular issues, metabolic abnormalities, and oxidative stress (OS) significantly contribute to its development^2,3^. Evaluating the incidence and prevalence rate of DPN is challenging, and the discrepancies in the reported prevalence of DPN among several countries are mainly attributed to variations in diagnostic standards between studies, differences in study populations, including disease duration and clinical settings, as well as regional and temporal variations^4^. According to epidemiology, up to 50% of diabetic people have DPN^5^. The overall prevalence of DPN from Europe and the United States approximately from 6% to 51% based on the population studied^6^. In contrast, the prevalence of DPN was found to be 71.2% in China^7^, and from 18.8% to 61.9% in India^8^. However, the percentage in Tanzania was 72.2%^9^. Nevertheless, in the Middle East, the general DPN rate was 53.7%^10^. In Sudan, the prevalence was 42%^11^. Current literature carried out in Saudi Arabia displays that 39% of diabetic individuals have DPN^12^. In Egypt, based on the national data, the development of DPN is about more than 60% of diabetic’s patients^13^.

Numerous genetic variations in candidate genes have been examined as potential risk factors, with many linked to several mechanisms such as the generation of reactive oxygen species (ROS)^14^. Os, which is an essential pathophysiological pathway of DPN, has gained considerable attention. The excessive generation of ROS, coupled with a reduction in antioxidant defenses, leads to disrupted redox homeostasis, which subsequently results in OS and ROS-mediated damage to critical biomacromolecules, including DNA in DPN^15,16^. DNA repair genes play a crucial role in maintaining genome integrity by restoring intact DNA through various mechanisms like nucleotide excision repair, double-strand break, and repair base excision repair (BER)^17,18^.

The X-ray cross complementary repair gene 1 (XRCC1), a member of the BER pathway, is responsible for repairing defects in DNA single-strand breaks and facilitating sister chromatid exchange, which occurs following exposure to ROS, alkylating agents, or ionizing radiation (IR)^19^. It is located on the long arm of human chromosome 19q13.2–13.3.3^20^. The gene comprises 17 exons and encodes a 70 kDa protein (approximately 633 amino acids). XRCC1 protein organizes the BER pathway, serving as a scaffold for specific repair enzymes and facilitating subsequent enzymatic processes^21^. The deactivation of the XRCC1 gene through genetic alterations leads to a decline in genetic stability, accompanied by an increase in the occurrence of spontaneous and/or induced chromosome deletions and translocations and as a result influence the risk of T2DM and its vascular complications^22^. Recent studies on XRCC1 gene polymorphisms focus primarily on three nonsynonymous SNPs: Arg399Gln (rs25487), Arg194Trp (rs1799782), and Arg280His (rs25489) SNPs^23^. These SNPs, especially Arg399Gln (rs25487), Arg194Trp (rs1799782) SNPs, could influence susceptibility to diabetes, through reducing DNA repair capacity in diabetic patients, resulting in reduced oxidative DNA damage repair and heighted sensitivity to OS^24–26^. Numerous studies indicate that polymorphisms in the XRCC1 gene may play a role in the pathogenesis of various cancer types^27–29^; however, research on T2DM and its complications, particularly DPN, remains relatively limited^26,30^. Moreover, given the high prevalence of T2DM and DPN in the Egyptian population^31,32^, and yet no previous studies regarding XRCC1 gene polymorphisms in our cohort, this study aims to enhance the understanding of DPN pathogenesis by investigating the roles of XRCC1 Arg399Gln and Arg194Trp SNPs in susceptibility to T2DM and DPN among Egyptian individuals. We aimed to develop accurate predictive models using advanced machine learning algorithms, addressing existing research gaps and identifying novel genetic and clinical risk factors. Furthermore, we examined the relationship between these SNPs and the severity of DPN using established scoring systems. This study integrates genetic predisposition, clinical factors, and ML-driven insights to facilitate more personalized approaches to the early detection, prevention, and targeted treatment of DPN.

## Subjects and methods

### Study patients selection

This study included a total of 732 participants, comprising 503 patients diagnosed with Type 2 Diabetes Mellitus (T2DM) and 229 healthy controls. The diabetic patients were divided into two groups: group 1:247 patients with uncomplicated T2DM, group 2: 256 patients with T2DM complicated by Diabetic Polyneuropathy (DPN). The control group consisted of 229 apparently healthy individuals with normal glucose metabolism and matched by age and sex. The overall case-to-control ratio was approximately 2.2:1 (503 cases to 229 controls). Among the cases, the ratio of DPN complicated to uncomplicated T2DM was approximately 1.04:1 (256 DPN to 247 uncomplicated).

Patients were enrolled sequentially during routine visits at diabetes outpatient clinics, diabetic neuropathy clinic, and inpatient wards of Zagazig Specialized Medical Hospital, Zagazig University, Zagazig, Egypt. Patients with T2DM were diagnosed according to the classification and criteria established by the American Diabetes Association (ADA)^33^. The individuals in the control group were selected from those attending general check-ups at the hospital. Patients involving autonomic or peripheral neuropathies due to factors other than diabetes, advanced-stage peripheral arterial disease, type 1 diabetes mellitus (T1DM), and severe comorbidities such as cancers, recent cardiovascular diseases, heart failure, advanced liver disease, or renal failure were excluded from the study. Additionally, cases administrating long-term immunomodulatory or immunosuppressive treatment were excluded from the study. Data collection involved collecting detailed personal information, such as age, sex, smoking status, and disease history, through discussions with all participants. In addition, clinical investigations such as comorbidity, blood glucose level at the time of neurological examination, calculated body mass index (BMI), the circumference of the waist, and disease duration were obtained from outpatient medical records. Routine clinical-laboratory data such as fasting blood glucose (FBG) level, lipid profile, and Hemoglobin A1C (HbA1c) were retrieved from the medical record sheet. By using protocols of calibrated devices and standardized measurement, the body weight and height were recorded on the verge of 0.1 kg and 0.1 cm, respectively^34^. At the level of the iliac crest, waist circumference (WC) was recorded to the nearest 0.1 cm while the participant was in standing position and breathing normally^35^. Following a minimum fasting period of 11 h, blood samples were obtained to evaluate serum cholesterol, triglycerides, lipoproteins, fasting glucose levels, and The Hemoglobin A1c (HbA1c) as detailed in aprior reports^35,36^.

Nerve conduction studies (NCS) were performed in the electrophysiology unit at the Neurology Department- Zagazig Specialized Medical Hospital using an EMG Machine (Nicolet, Natus Neurology, USA). Furthermore, neuropathy was assessed in diabetic patients according to the Toronto Clinical Neuropathy Scoring System (TCNS) and Modified Neuropathy Disability Score (NDS).

The Institutional Ethics Committee of the Faculty of Medicine of Zagazig University in Egypt approved the study protocol. In addition, the study adhered to the ethical criteria of the Declaration of Helsinki (ZU-IRB #496/25 Aug-2024). Prior to participation in the study, each participant provided written informed consent.

## Methods

### DNA isolation and molecular genetic analysis

The QIAamp DNA Blood Mini kit (Cat-No: #51104; Qiagen, Valencia, California, USA) was used to isolate the genomic DNA from collected samples with anticoagulant (EDTA) according to the manufacturer’s information. A NanoDrop spectrophotometer (ND1000; NanoDrop Technologies, Wilmington, Delaware, USA) was used to measure the purity and concentration of isolated DNA and frozen at −80 °C until genetic testing.

**Fig. 1:**
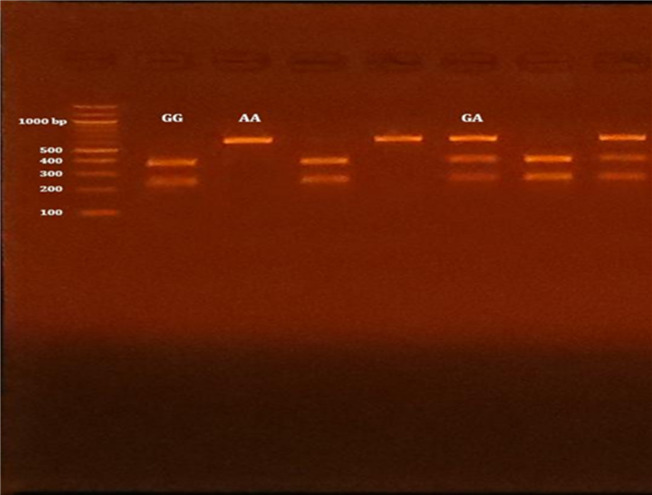
Agarose gel electrophoresis presenting the PCR amplification outcomes of Arg399Gln (rs25487) SNP in XRCC1 gene. M: DNA marker (100 bp); Lines 2,4,7: GG gentype (375 bp and 240 bp); lines 3,5: PCR products ans AA genotypes (615 bp); lines 6,8: GA genotypes (615 bp, 375 bp, abd 240 bp).

**Fig. 2:**
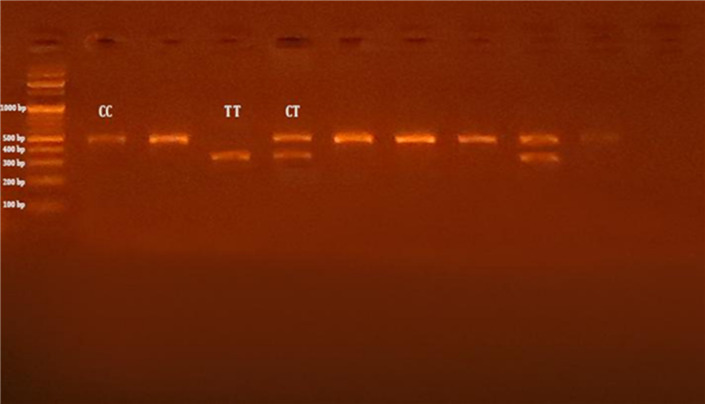
Agarose gel electrophoresis presenting the PCR amplification outcomes of Arg194Trp (rs1799782) SNP in XRCC1 gene. M: DNA marker (100 bp); Lines 2,3,6,7,8,10: PCR products and CC gentypes (485 bp); line 4: TT genotypes (396 bp and 89 bp); lines 5,9: CT genotypes (485 bp, 396 bp, abd 89 bp). The small fragment of 89 bp was invisible in the gel.

The polymerase chain reaction-restriction fragment length polymorphism (PCR-RFLP) analysis was conducted to detect polymorphic sites in the XRCC1 gene using specific primer pairs for Arg399Gln (rs25487) SNP^37^ and Arg194Trp (rs1799782) SNP^38^. In brief, considering details on the determination of the SNP alleles, successful amplification of the Arg399Gln (rs25487) and Arg194Trp (rs1799782) SNPs, were demonstrated by the presence of PCR amplified products with sizes of 615 bp, and 485 bp, respectively. Regarding Arg399Gln (rs25487) SNP, after digestion by MpsI restriction enzyme, the PCR-RFLP results were the Arg/Arg genotype (G/G wild type homozygous) (digested product) (375 bp and 240-bp), the Arg/Gln genotype (G/A heterozygous) (615 bp, 375 bp, and 240 bp), and the Gln/GLn genotype (A/A mutant homozygous) (undigested product) (615 bp) (Fig. 1). In the case of Arg194Trp (rs1799782) SNP, after digestion by PvuII restriction enzyme, the PCR-RFLP results were the Arg/Arg genotype (C/C wild type homozygous) (undigested product) (485 bp), the Arg/Trp genotype (C/T heterozygous) (485 bp, 396 bp, and 89 bp), and the Trp/Trp genotype (T/T mutant homozygous) (digested product) (396 bp, and 89 bp) (Fig. 2).

For carrying out the first step of RFLP technique (PCR reaction), template DNA was amplified to generate PCR products for both SNPs in a total volume of 20 µL consisting of 10 µL Taq PCR Master Mix (Thermo Fisher Scientific, Massachusetts, USA), 1.5 µL of each of forward and reverse primers (10 pmol/µL), 4.5 µL sterile deionized water, and 2.5 µL of template DNA (about 30ng). PCR Cycling conditions were performed in a thermal cycler (Biometra, Gottingen, Germany) as follows: For the Arg399Gln SNP, the protocol includes an initial denaturation at 94 °C for 5 min, followed by 40 cycles consisting of denaturation at 94 °C for 30 s, annealing at 57 °C for 30 s, and extension at 72 °C for 30 s, concluding with a final extension at 72 °C for 7 min. For the Arg194Trp SNP, the protocol involved an initial denaturation at 94 °C for 5 min, followed by 35 cycles consisting of denaturation at 94 °C for 30 s, annealing at 59 °C for 30 s, and extension at 72 °C for 30 s, concluding with a final extension of 7 min at 72 °C. Following the PCR reaction, for performing the second step of RFLP technique, the PCR products underwent digestion with FastDigest MpsI and PvuII restriction enzymes for Arg399Gln (rs25487) and Arg194Trp (rs1799782) SNPs, respectively (New England Biolabs, Massachusetts, USA) and were incubated for 15 min at 37 °C. The specific fragments were separated on a 1.5% agarose gel, stained with Ethidium Bromide, and visualized under UV light using a gel documentation system.

### Statistical analyses

First, data were screened for outliers using boxplots and assessed for missing values, which were absent. Numeric data are presented as mean ± standard deviation (SD), whereas categorical variables are reported as frequencies (%). Hardy-Weinberg equilibrium (HWE) was evaluated using Pearson’s χ² test. ANOVA (for three-group comparisons) and independent *t*-tests (for two-group comparisons) were applied to normally distributed data. Skewed or heteroscedastic data were analyzed with a generalized linear gamma model incorporating a log-link function. Genetic associations of XRCC1 Arg399Gln and XRCC1 Arg194Trp polymorphisms were evaluated under codominant, dominant, overdominant, and recessive genetic models, with the optimal model selected based on the lowest Akaike Information Criterion (AIC) value. Genotype and allele frequencies were computed, and univariate logistic regression was employed to estimate odds ratios (ORs) with 95% confidence intervals (CIs). Haplotype analysis was performed using the SHEsis online platform http://shesisplus.bio-x.cn/SHEsis.html, based on the expectation–maximization (EM) algorithm.

### Machine learning (ML) models

Data were randomly partitioned (80:20 ratio) into training and testing sets. Hyperparameter optimization was performed via grid search using the XGBoost model (hyperparameters are provided in the supplementary materials), and 10-fold cross-validation was implemented to mitigate overfitting. Feature selection was conducted using Random Forest, while XGBoost was utilized to predict DPN risk factors. SHAP (SHapley Additive exPlanations) analysis enhanced model interpretability by identifying and ranking key predictors. ML models were implemented in R (R Core Team)^39^ using the *randomForest* and *xgboost* packages.

## Results

###  Demographical and clinical-laboratory data of studied groups

As shown in Table 1, patients with DPN had a significantly higher age than those in the uncomplicated T2DM and control groups (*P* < 0.05). Fasting blood glucose (FBG) levels were also significantly higher in the DPN group compared with both the uncomplicated T2DM and control groups (*P* < 0.001). All clinical-laboratory data were also significantly elevated in the DPN group compared to both other groups except high-density lipoprotein (HDL) levels were at their lowest in the DPN group. Disease duration was notably longer in the DPN group than in the uncomplicated group.

**Table 1:** Demographical and clinico-laboratory data of studied groups.

	T2DM Patients (*N* = 340)			
	T2DM group complicated with DPN (*N* = 256)	Uncomplicated T2DM group (*N* = 247)	Control group (*N* = 229)	*P*-value
Demographical Data				
Age^a^	50.00 ± 0.48 ^a^	44.3 ± 0.44 ^b^	42.3 ± 0.43 ^c^	< 0.05
Gender^b^ M/F	120/136	110/137	126/103	*P* = 0.056
Smoking/Y/N	46/210	38/209	53/176	*P* = 0.08
Clinico-laboratory Data				
FBG (mg/dl)^a^	233.60 ± 1.84^a^	115.60 ± 0.92^b^	82.3 ± 0.68^c^	< 0.001
HBAIC^a^	9.20 ± 0.08	7.03 ± 0.07	----	< 0.001
BMI (Kg/m^2^)^a^	29.9 ± 0.25^a^	23.6 ± 0.20^c^	24.6 ± 0.22 ^b^	< 0.05
TG^a^	239.00 ± 1.64^a^	190.00 ± 1.33^b^	181.00 ± 1.32^c^	< 0.05
LDL cholesterol ^a^	176.7 ± 0.96^a^	118.00 ± 0.65^b^	91.7 ± 0.52c	< 0.05
HDL cholesterol ^a^	37.40 ± 0.23^b^	47.00 ± 0.29^a^	47.6 ± 0.31^a^	< 0.05
Duration of disease ^a^	10.08 ± 0.21	4.32 ± 0.09		< 0.0001
TCNS	10.28 ± 0.22			
NDS	7.11 ± 0.15			

Abbreviations: DPN, Diabetic peripheral neuropathy; BMI, Body mass index; FBG, Fasting blood glucose; LDL, Low density lipoprotein; HDL, High density lipoprotein; TG, Triglyceride; HBAIC, Hemoglobin A1C; TCNS, Toronto Clinical Neuropathy Score; NDS, Neuropathy Disability Score. ^a^Data were reported as mean ± SD. ^b^ Data were reported as number and percentage(%).

^abc^Means carrying different superscripts within same row are statistically different.

### Genetic model analysis of the association between XRCC1 SNPs and the risk of DPN among studied groups (adjusted for age, sex, and smoking status)

Both SNPs were in Hardy-Weinberg equilibrium (HWE, *P* > 0.05) among T2DM patients, including both complicated and uncomplicated groups, as well as in the control group. The genotyping of both SNPs is presented in Figs. 1 and 2, respectively. As shown in Table 2, illustrates that the codominant model most effectively accounted for the association between the XRCC1 Arg 399 Gln SNP and the risk of DPN [P^1^ < 0.0001], as indicated by the lowest AIC values (AIC^1^ = 511.1) when comparing the DPN group to control group, indicating that the G/A and A/A genotypes are associated with an approximate 2-fold and 8-fold increase in DPN risk, respectively. Compared to the uncomplicated T2DM group, the DPN group exhibited significant associations across the codominant, dominant, recessive, and overdominant models, with the codominant model [P^3^ = 0.00009, and AIC^3^ = 614.9] demonstrating the best fit. Under this model, the G/A and A/A genotypes were associated with an approximate 2-fold and 4-fold increase in DPN risk, respectively. No significant associations were observed in any genetic model when comparing the uncomplicated T2DM group to the control group. The XRCC1 Arg399Gln A allele was associated with a nearly threefold increase in the risk of DPN [P^1^ < 0.00001] compared to controls and a twofold increase [P^3^ = 0.00004] compared to the uncomplicated T2DM group. These findings indicate that the observed associations are independent of age, sex, and smoking, which were adjusted for to control confounding and ensure that the genetic effects reflect true associations with DPN risk.

**Table 2:** Genetic model analysis of the association between XRCC1 Arg399Gln SNP and the risk of DPN among studied groups (adjusted for age, sex and smoking).

Model	Genotype	complicated	Uncomplicated	control	OR (95% CI)^1^ Complicated vs. control	OR (95% CI)^2^ UnComplicated vs. control	OR (95% CI)^3^ Complicated vs. uncomplicated	*P*-value^1^	AIC^1^	*P*-value^2^	AIC^2^	*P*-value^3^	AIC^3^
**Codominant**	**G/G**	81 (31.6%)	107 (43.3)	113 (49.3%)	**1.0 (ref.)**	**1.0 (ref.)**	**1.0 (ref.)**	< 0.0001	**511.1**	0.24	652.9	0.00009	**614.9**
	**G/A**	133 (52%)	121(49.0)	99 (43.2%)	2.26 (1.42–3.62)	1.36 (0.92–1.99)	1.65 (1.08–2.51)						
	**A/A**	42 (16.4%)	19 (7.7)	17 (7.4%)	**8.04 (3.65–17.68)**	**1.47 (0.71–3.07)**	**4.10 (2.09–8.03)**						
**Dominant**	**G/G**	81 (31.6%)	107 (43.3)	113 (49.3%)	**1.0 (ref.)**	**1.0 (ref.)**		< 0.0001	520.9	0.09	**651**	0.001	621.1
	**G/A-A/A**	175 (68.4%)	140 (56.7)	116 (50.7%)	2.79 (1.79–4.37)	1.37 (0.95–1.99)	1.94(1.29–2.90)						
**Recessive**	**G/G-G/A**	214 (83.6%)	228 (92.3)	212 (92.6%)	**1.0 (ref.)**	**1.0 (ref.)**	**1.0 (ref.)**	< 0.0001	521.3	0.52	653.3	0.0003	618.4
	**A/A**	42 (16.4%)	19 (7.7)	17 (7.4%)	4.95 (2.40- 10.19)	1.26 (0.62–2.55)	3.03(1.63–5.62)						
**Overdominant**	**G/G-A/A**	123 (48%)	126 (51)	130 (56.8%)	**1.0 (ref.)**	**1.0 (ref.)**	**1.0 (ref.)**	0.11	539.6	0.18	652	0.0004	631.1
	**G/A**	133 (52%)	121 (49)	99 (43.2%)	1.41 (0.93–2.14)	1.28 (0.89–1.86)	1.15(0.79–1.68)						
**Alleles**	**Allele G**	295 (57.62)	335 (67.81)	325(70.96)	**1.0 (ref.)**	**1.0 (ref.)**	**1.0 (ref.)**						
	**Allele A**	**217 (42.30)**	159 (32.19)	**133(29.04)**	2.59 (1.84–3.64)***	1.18 (0.89–1.57)^NS^	1.89 (1.39–2.57)***	< 0.0001		0.27		0.00004	

AIC: Akaike Information Criterion; ref.: reference category; OR: odds ratio; CI: confidence interval; significant difference:*P < 0.05,***P < 0.001; NS: non-significant difference: *P* > 0.05; P-value^1,2,and3^ of ORs and AIC^1,2, and3^ are for complicated vs. control, uncomplicated vs. control, and complicated vs. uncomplicated, respectively. P-values were calculated by logistic analysis after adjusting for age, gender, smoking status.

Table 3 illustrates that the dominant genetic model most effectively accounted for the association between the XRCC1 Arg194Trp SNP and the risk of DPN, as indicated by the lowest AIC values [AIC^1^ = 531.0, AIC^2^ = 648.2, AIC^3^ = 626.5] when comparing the DPN group to both the uncomplicated T2DM and control groups. The C/T or T/T genotypes were associated with a significantly increased risk of DPN by 2-fold compared to controls [P^1^ = 0.0008], by 1.57-fold compared to the uncomplicated T2DM group [P^3^ = 0.02], and by 1.57-fold compared to controls in the uncomplicated group [P^2^ = 0.018]. The results suggest that possessing a single copy of the T allele (C/T or T/T genotypes) is adequate to elevate the risk of DPN. Furthermore, allele analysis demonstrated a significant correlation between the T allele and susceptibility to DPN, indicating a 1.78-fold increased risk for complicated DPN (P^1^ = 0.001) and a 1.48-fold increased risk for uncomplicated DPN [P^2^ = 0.01]. The findings indicate that the T allele of XRCC1 Arg194Trp may function as a genetic risk factor for the development of DPN.

**Table 3:** Genetic model analysis of the association between XRCC1 Arg194Trp SNP and the risk of DPN among studied groups (adjusted for age, sex and smoking).

Model	Genotype	complicated	Uncomplicated	control	OR (95% CI)^1^ Complicated vs. control	OR (95% CI)^2^ UnComplicated vs. control	OR (95% CI)^3^ Complicated vs. uncomlicated	*P*-value^1^	AIC^1^	*P*-value^2^	AIC^2^	*P*-value^3^	AIC^3^
Codominant	**C/C**	**116 (45.31)**	**114 (46.15)**	**130 (56.77)**	**1.0 (ref.).**	**1.0 (ref.).**	**1.0 (ref.)**	**0.003**	**532.7**	**0.057**	**650**	**0.07**	**628.2**
	**C/T**	**122 (47.66)**	**117(47.37)**	**91 (39.74)**	**1.99 (1.28–3.10)**	**1.54 (1.04–2.27)**	**1.63 (1.08–2.47)**						
	**T/T**	**18 (7.03)**	**16 (6.48)**	**8 (3.49)**	**2.57 (1.03–6.46)**	**1.88(0.76–4.61)**	**1.27 (0.59–2.71)**						
Dominant	**C/C**	**116 (45.30)**	**114 (46.2)**	**130 (56.8)**	**1.0 (ref.)**	**1.0 (ref.)**	**1.0 (ref.)**	**0.0008**	**531.0**	**0.018**	**648.2**	**0.02**	**626.5**
	**C/T-T/T**	**140 (54.70)**	**133 (53.8)**	**99 (43.2)**	**2.06 (1.34–3.16)**	**1.57 (1.08–2.29)**	**1.57 (1.06–2.34)**						
Recessive	**C/C-C/T**	**238 (93.00)**	**231 (93.5)**	**221 (96.5)**	**1.0 (ref.)**	**1.0 (ref.)**	**1.0 (ref.)**	**0.16**	**540.3**	**0.308**	**652.7**	**0.98**	**631.6**
	**T/T**	**18(7.00)**	**16 (6.5)**	**8 (3.50)**	**1.86 (0.76–4.54)**	**1.57 (0.65–3.80)**	**0.99 (0.48–2.05)**						
Overdominant	**C/C-T/T**	**134(52.30)**	**130 (52.6)**	**138(60.30)**	**1.0 (ref.)**	**1.0 (ref.)**	**1.0 (ref.)**	**0.007**	**535.1**	**0.05**	**650**	**0.02**	**626.6**
	**C/T**	**122 (47.7)**	**117 (47.4)**	**91 (39.7)**	**1.79 (1.16–2.74)**	**1.46 (0.99–2.14)**	**1.57 (1.06–2.34)**						
Alleles	**C**	**354 (69.14)**	**345(69.84)**	**351 (76.64)**	**1.0 (ref.)**	**1.0 (ref.)**	**1.0 (ref.)**						
	**T**	**158 (30.86)**	**149 (30.16)**	**107 (23.36)**	1.78 (1.25–2.54)***	1.48 (1.09–2.02)**	**1.33 (0.97–1.83)** ^**NS**^	**0.001**		**0.01**		**0.07**	

AIC: Akaike Information Criterion; ref.: reference category; OR: odds ratio; CI: confidence interval; significant difference:*P < 0.05,***P < 0.001; NS: non-significant difference: *P* > 0.05; P-value^1,2,and3^ of ORs and AIC^1,2, and3^ are for complicated vs. control, uncomplicated vs. control, and complicated vs. uncomplicated, respectively. P-values were calculated by logistic analysis after adjusting for age, gender, smoking status.

### Haplotype analysis

Haplotype analysis supports the genetic associations between XRCC1 SNPs and the risk of DPN (Table 4). The ‘A-T’ haplotype was associated with a 1.28-fold increase in DPN susceptibility relative to the reference ‘G-C’ haplotype [P = 0.000001], reflecting the risk attributed to the Arg399Gln variant. In addition, the ‘A-C’ and ‘G-T’ haplotypes demonstrated a significant association with an increased risk of T2DM in patients without DPN [P = 0.0027, and P < 0.00001, respectively]. The ‘A-T’ haplotype was identified as a significant predictor of DPN risk [P < 0.00001], while ‘G-T’ and ‘A-C’ haplotypes exhibited protective effects [*P* = 0.0389, and *P* = 0.0001, respectively] when comparing DPN group to the uncomplicated group. These results emphasize that haplotype-based analysis captures multi-locus interactions that single SNP analyses may overlook. Although the T and A alleles individually conferred risk, their combined presence within the G–T and A–C haplotypes likely exerted compensatory or non-additive effects, resulting in a net protective influence. This underscores the role of linkage disequilibrium and inter-allelic interactions in modulating genetic susceptibility to DPN. These findings highlight the pivotal role of XRCC1 haplotypes in shaping susceptibility to T2DM and its complications, particularly DPN.

**Table 4:** Impact of haplotypes of XRCC1 Arg399Gln (G/A) and XRCC1 Arg194Trp (C/T) on T2DM and DPN risk.

	frequency	OR	95% C.I.	*P*-value
	Control Vs. complicated			
G-C	0.5323		Reference haplotype	
A-C	0.1945	1.09	(0.99–1.19)	0.06
A-T	0.1664	1.28	(0.91–1.12)	0.000001
G-T	0.1068	1.002	(0.84–1.05)	0.97
	Control Vs. uncomplicated			
G-C	0.5134		Reference haplotype	
A-C	0.2177	1.12	(1.05–1.26)	0.0027
A-T	0.089	0.97	(0.84–1.12)	0.7052
G-T	0.1799	1.23	(1.12–1.36)	< 0.00001
	Complicated Vs. uncomplicated			
G-C	0.4582		Reference haplotype	
A-C	0.2366	0.90	(0.83–0.98)	0.0389
A-T	0.1372	1.34	(1.19–1.49)	< 0.00001
G-T	0.168	0.84	(0.76–0.91)	0.0001

### Genotypic distribution of XRCC1 SNPs based on TCNS and NDS and their association with severity of DPN

Table 5 displays the chi-square test results evaluating the association between the genotypic distribution of both SNPs and the severity of DPN, as measured by TCNS and NDS scores. The results demonstrate that the G/G and C/C wild-type genotypes were predominantly found in mild cases, while the A/A (and combined G/A + A/A) genotypes for XRCC1 Arg399Gln SNP and the C/T (and combined C/T + T/T) genotypes for XRCC1 Arg194Trp SNP were most frequently linked to severe DPN cases. Table 6 presents the evaluation of the XGBoost algorithm, indicating that TCNS (96%) demonstrates superior predictive performance compared to NDS (90%) in the assessment of DPN severity. TCNS demonstrated significantly higher accuracy, sensitivity, specificity, precision, F1-score, and AUC, highlighting its enhanced effectiveness in predicting DPN severity.

**Table 5:** Genotypic distribution of XRCC1 Arg399Gln and Arg194Trp SNPs based on TCNS and NDS and their association with severity of DPN.

SNPs	TCNS				NDS				
XRCC1 Arg3991Gln (G/A				*P*-value	XRCC1 Arg3991Gln (G/A			*P*-value	
	Mild	Moderate	Severe			Mild	Moderate	Severe	
G/G	54 (66.7%)	22 (27.2%)	5 (6.2%)	< 0.0001**	G/G	54 (66.7%)	17 (21.0%)	10 (12.3%)	< 0.0001**
G/A	36 (27.1%)	50 (37.6%)	47 (35.3%)		G/A	36 (27.1%)	36 (27.1%)	61 (45.9%)	
A/A	4 (9.5%)	14 (33.3%)	24 (57.1%)		A/A	4 (9.5%)	4 (9.5%)	34 (81.0%)	
G/A + A/A	40 (22.90%)	64 (36.57%)	71 (40.53%)	0.01*	G/A + A/A	40(22.85%)	40 (22.85%)	95 (54.29%)	< 0.0001**
XRCC1 Arg194Trp (C/T)				P-value	XRCC1 Arg194Trp (C/T)			P-value	
	Mild	Moderate	Severe			Mild	Moderate	Severe	
C/C	88 (75.9%)	28 (24.1%)	0 (0.0%)	< 0.00001	C/C	88 (75.9%)	28 (24.1%)	0	< 0.00001****
C/T	0	47 (38.5%)	75 (61.5%)		C/T	0	20 (16.4%)	102 (83.6%)	
T/T	6 (33.3%)	11 (61.10%)	1 (5.6%)		T/T	6 (33.3%)	9 (50.0%)	3 (16.7%)	
C/T + T/T	6(4.3%)	58(41.43%)	76 (54.29%)	< 0.00001	C/T + T/T	6 (4.3%)	29(20.71%)	105 (75%)	< 0.0001***

TCNS: Mild (5–8), Moderate (9–11), Severe (≥ 12); NDS: Mild (3–5), Moderate 6–8), Severe (9–10).

**Table 6:** Comparison between performance of TCNS and NDS in evaluating DPN based on XGBoost algorithm.

	TCNS			NDS		
	Mild	Moderate	Sever	Mild	Moderate	Severe
Overall Acuuracy	96%			90%		
Sensitivity	1.00	0.94	0.95	1.00	0.86	0.87
Spescificty	0.97	0.97	1.00	0.97	0.92	0.96
percision	0.93	0.94	1.00	0.93	0.80	0.95
F1 score	0.96	0.94	0.97	0.97	0.83	0.91
AUC	0.98	0.96	0.97	0.98	0.89	0.92

AUC: Area under the curve.

### Logistic regression analysis of risk factors and genetic polymorphisms in DPN

Logistic regression analysis identified several independent risk factors for DPN, including age, BMI, FBG, LDL, TG, G/A, and A/A genotypes of XRCC1 Arg399Gln SNP and C/T and T/T genotypes of XRCC1 Arg194Trp SNP. In contrast, sex and smoking showed no significant association with DPN risk (Table 7).

**Table 7:** Logistic regression analysis on the association between risk of DPN, and potential risk factors and genetic polymorphisms.

Variables		Odd ratio	95% CI	*P*-value
Age		1.19	1.15–1.23	< 0.000001
sex	Male	Ref.		
	Female	1.39	0.97–1.99	0.07^NS^
Smoking	Non smoker	Ref.		
	Smoker	0.73	0.47–1.13	0.15^NS^
BMI		1.67	1.54–1.84	< 0.000001
FBG		1.71	(1.67–1.76)	< 0.0001
LDL		2.55	(2.42–2.71)	< 0.0001
HDL		0.3	0.22–0.38	< 0.0001
TG		1.11	1.09–1.13	< 0.0001
XRCC1 Arg3991Gln (G/A)		1.87	1.28–2.76	0.001
AA		3.45	1.86–6.62	0.0001
XRCC1 Arg194Trp (C/T)		1.50	1.04–2.18	0.03
TT		2.52	1.09–6.35	0.03

Abbreviations: DPN, Diabetic peripheral neuropathy; BMI, Body mass index; FBG, Fasting blood glucose; LDL, Low density lipoprotein; HDL, High density lipoprotein; TG, Triglyceride; NS, Non significant. *P* < 0.05 is considered significant. *P* > 0.05 is considered non- significant.

**Table 8:** Summary of previous findings investigating XRCC1 Arg399Gln and Arg194Trp SNPs in T2DM and its complications across different populations.

Study (Year)	Population	SNPs	Disease	Genotypes	Assocaition observed
Kasznicki et al^46^.	Polish	Arg399Gln (G/A) SNP	T2DM	AA genotype	No association with T2DM.
Al-Musawi et al^47^.	Iraq	Arg399Gln (G/A) SNP	T2DM	AA genotype	No association with T2DM.
Yesil-Devecioglu et al^26^.	Turkish	Arg399Gln (G/A) SNP	T2DM and DN	AA genotype	Significant association with with T2DM and diabetic nephropathy (DN).
Narne et al^49^.	South Indian	Arg399Gln G/A) SNP)	T2DM and DR	AA genotype	Significant association with diabetic retinopathy (DR).
GÖKÇE et al^83^.	Turkish	Arg194Trp C/T) SNP)	T2DM	TT genotype	No association with T2DM.
Guo et al^84^.	Asian	Arg194Trp C/T) SNP)	CAD	TT genotype	Significant association with CAD.

T2DM : Type 2 diabetes mellitus, DN: Diabetic nephropathy, DR: Diabetic retinopathy. CAD: Coronary artery disease. No prior study has employed a machine learning approach to analyze the association between the investigated XRCC1 SNPs and DPN risk. In addition, a limited number of studies have addressed XRCC1 SNPs in DPN. Moreover, to date, no study conducted in Egypt study also has investigated this association.

### Machine learning approaches for key risk predictor selection

In addition to traditional statistical models such as logistic regression, we applied machine learning algorithms to further investigate the associations between key variables and DPN risk, overcoming the limitations of conventional approaches.

### Random forest (RF) analysis

**Fig. 3:**
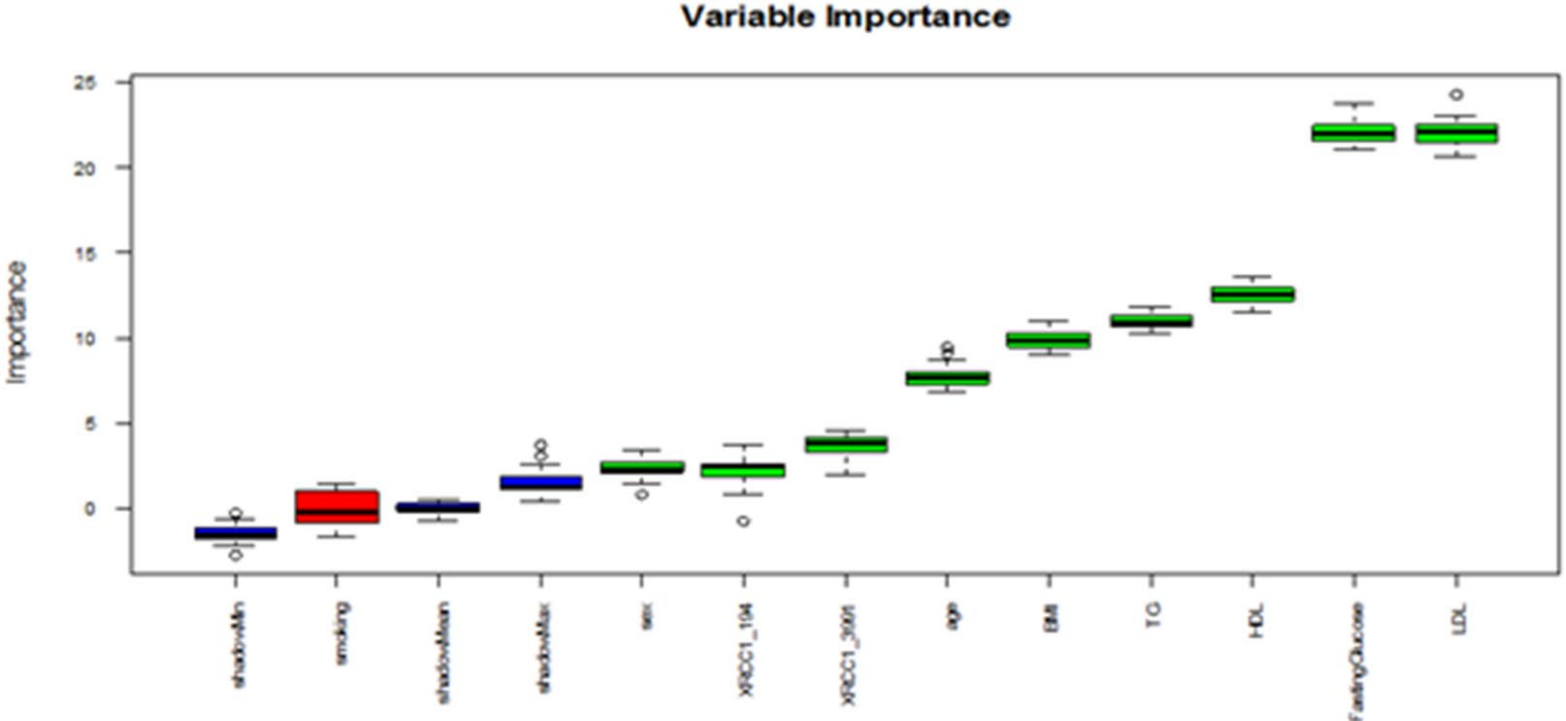
Variable importance plot generated by Bruta random forest analysis. ShadowMin, ShadowMean, and ShadowMax are non-sense variables, generated by randomly shuffling the original values of the predictors. The predictors above the shadow variables, marked by green boxplots, are important.

**Fig. 4:**
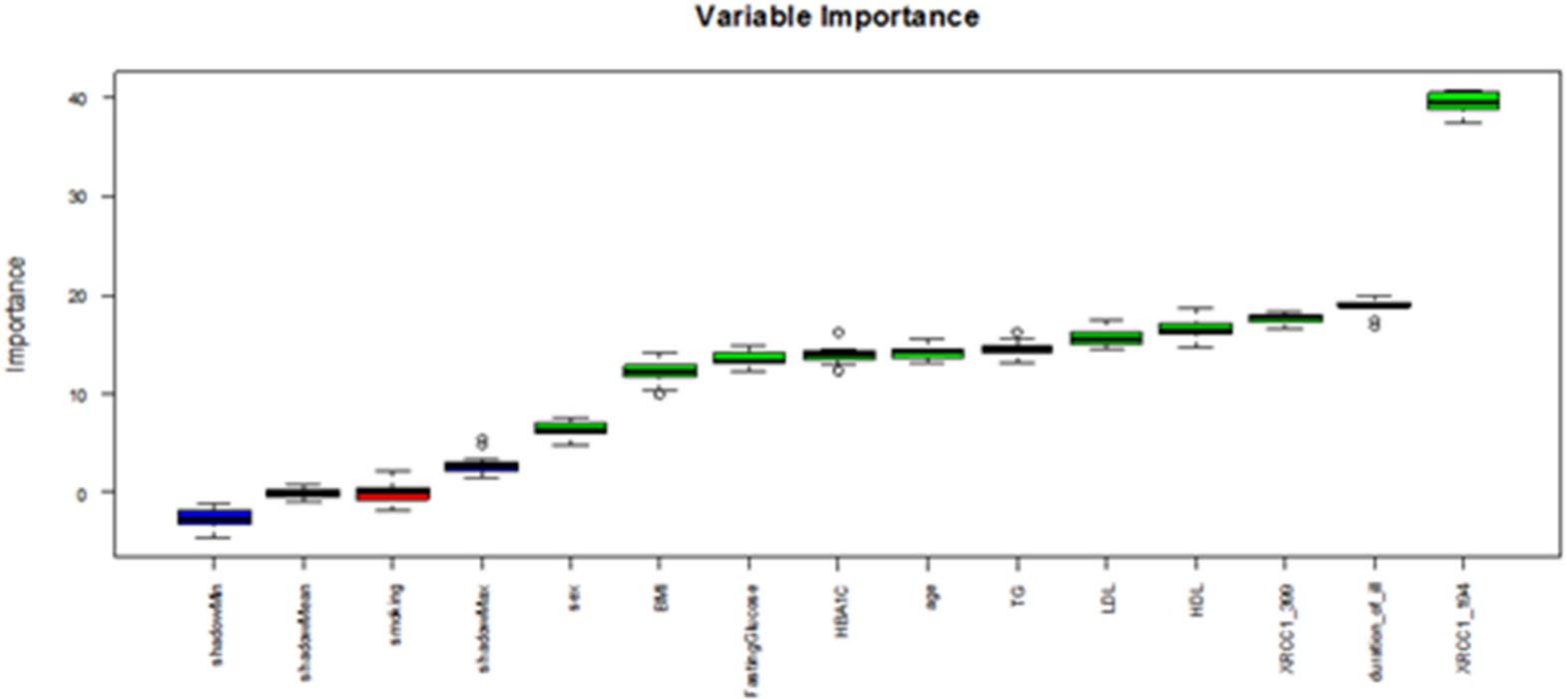
Variable importance plot for features used to predict complication based on TCNS. The predictors above the shadow variables, marked by green boxplots, are important.

RF demonstrated exemplary performance, achieving 98–100% accuracy in both training and test sets with optimized hyperparameters (ntree = 110, mtry = 7). Feature importance analysis identified LDL, FBG, HDL, TG, age, and BMI as the primary determinants of DPN risk, whereas smoking exhibited negligible impact. Notably, the XRCC1 Arg399Gln SNP demonstrated a more significant classification impact than the XRCC1 Arg194Trp SNP **(**Fig. 3**)**. Additionally, RF effectively identified XRCC1 Arg194Trp SNP, disease duration, and XRCC1 Arg399Gln SNP as significant factors influencing DPN severity **(**Fig. 4**)**, highlighting the interaction between genetic predisposition and clinical variables in disease progression.

### XGBoost model insights

**Fig. 5:**
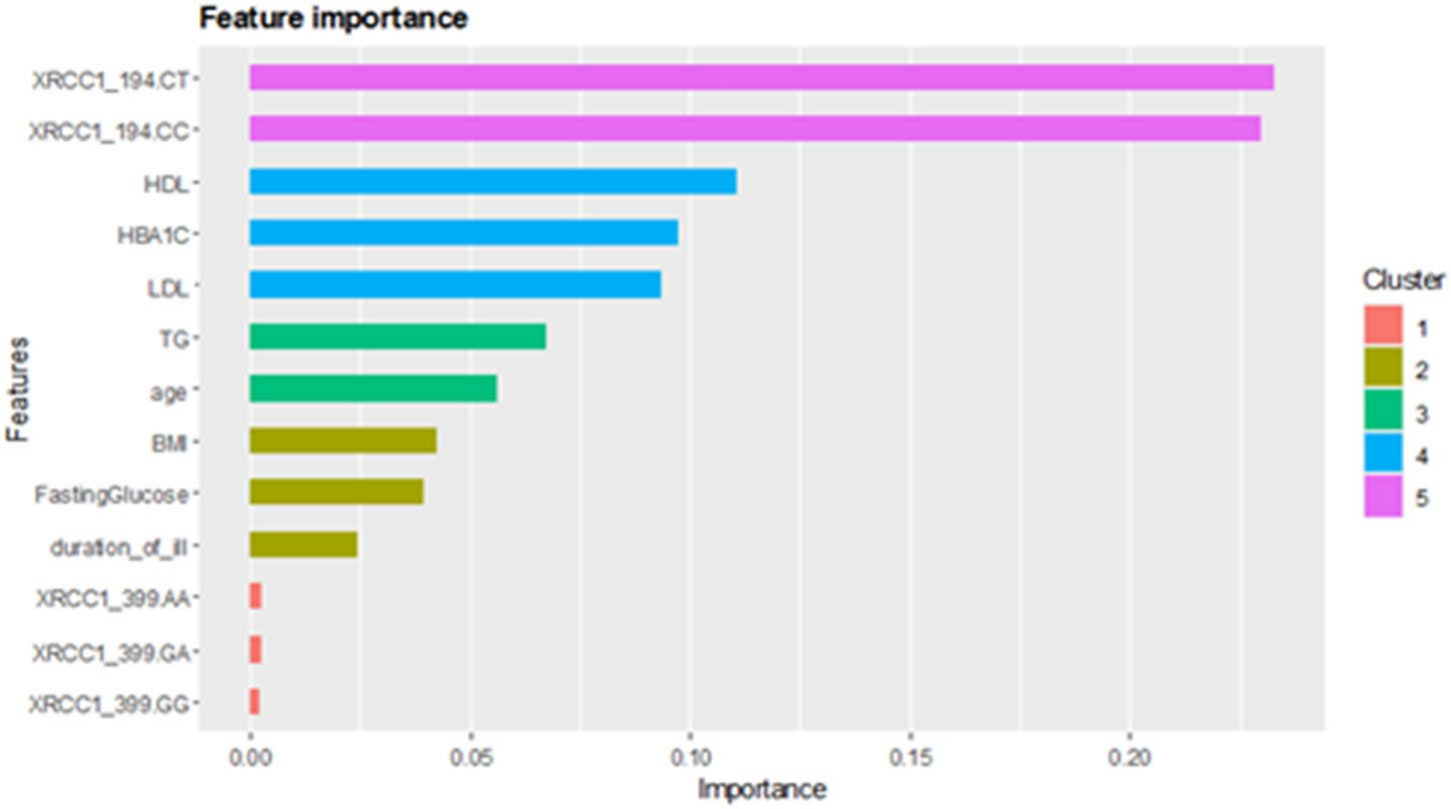
Variable importance of XGboost, with clustering features according to their closeness into one cluster.

Utilizing RF-selected features, XGBoost identified XRCC1 Arg194Trp SNP genotypes (C/T, C/C) as the most significant predictors of DPN, followed by biochemical markers (HDL, HbA1c, LDL). The XRCC1 Arg399Gln SNP genotypes (A/A, G/A, G/G) improved prediction accuracy **(**Fig. 5**)**, underscoring the significant influence of genetic and metabolic factors in DPN development.

### SHAP (SHapley additive exPlanations) analysis for feature interpretation

**Fig. 6:**
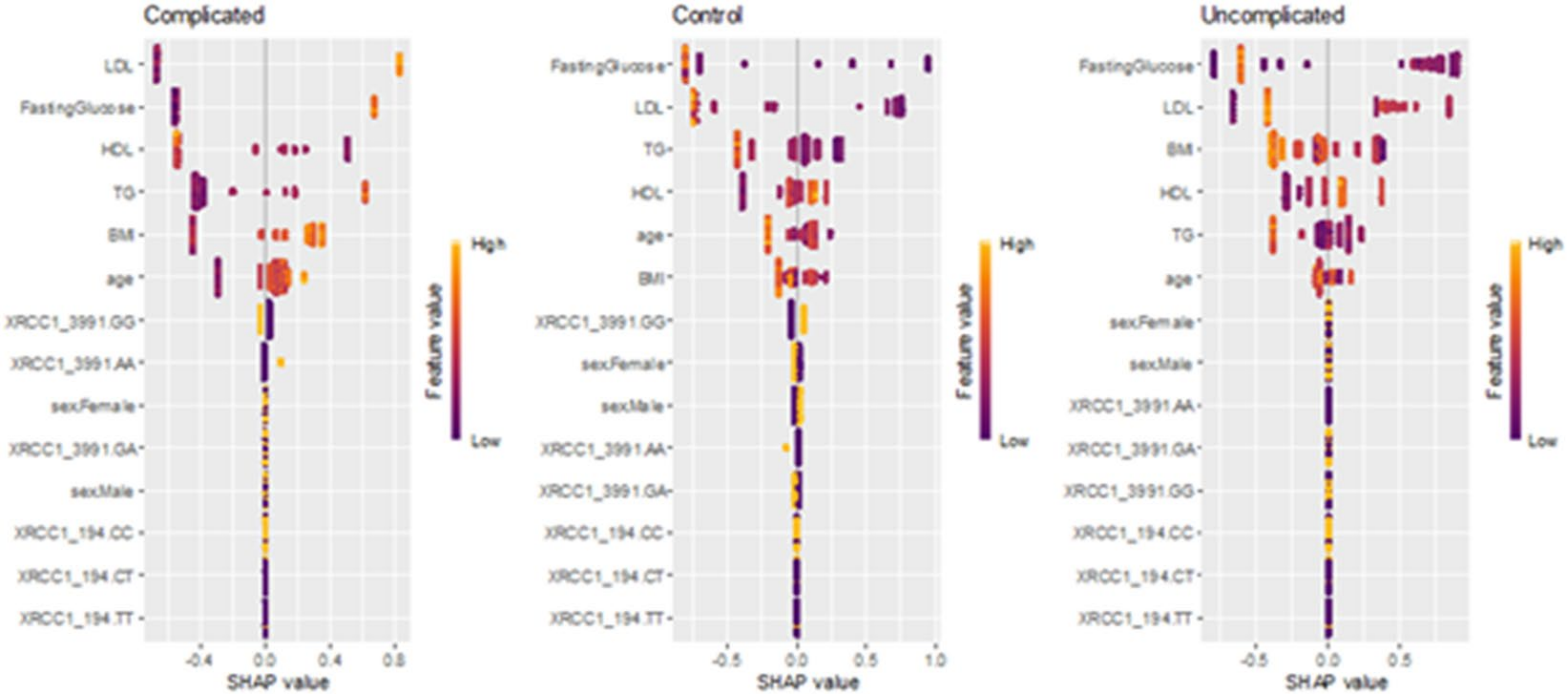
SHAP values for the XGBoost model. Each point represents a sample. The higher the SHAP value the higher the risk of DPN, and vice versa.

**Fig. 7:**
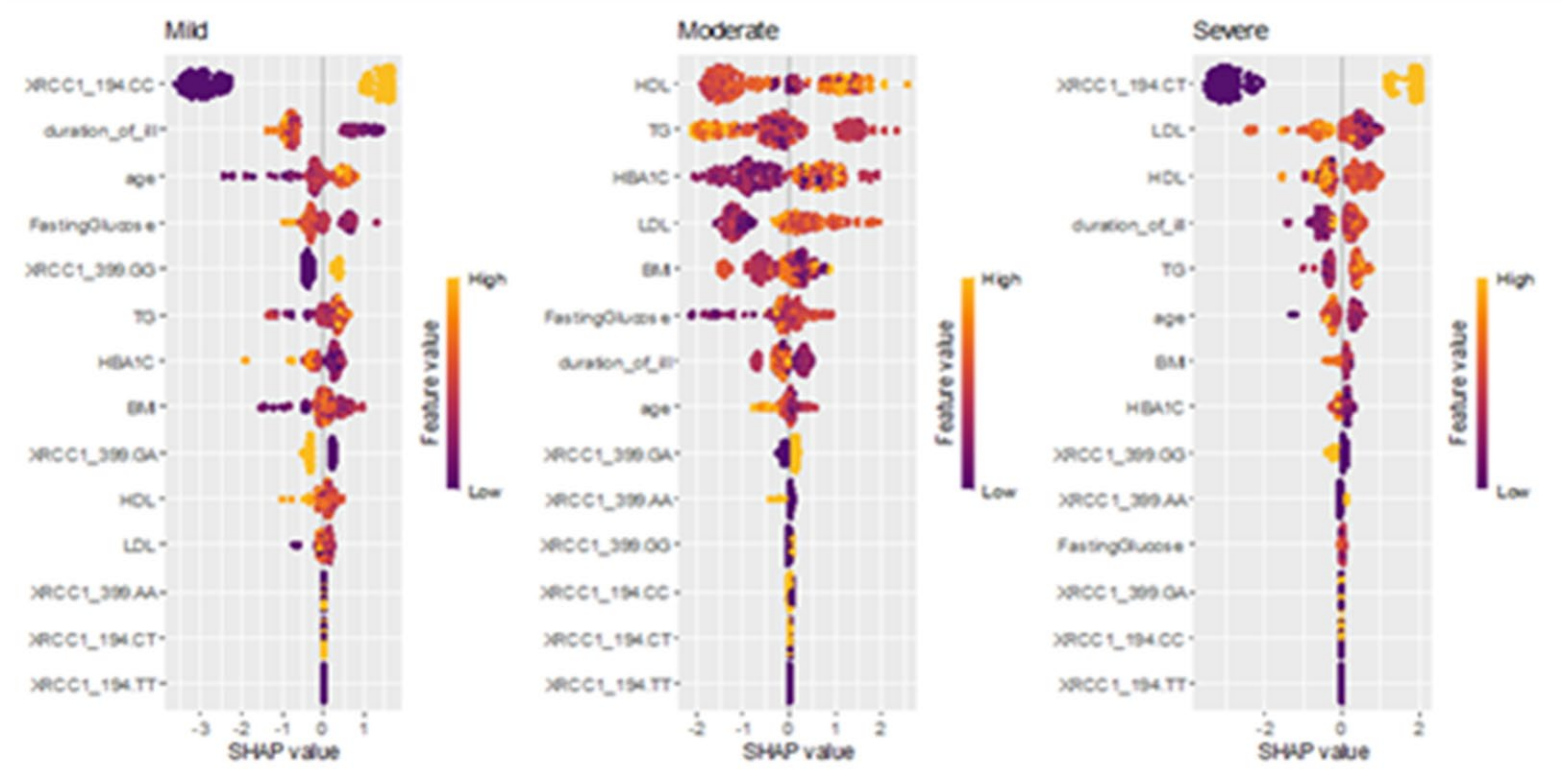
SHAP values for the XGBoost model of TCNS.

SHAP analysis, an additive explanatory framework quantifying both the magnitude and directionality of effects, was applied to gain a deeper understanding. Figure 6 demonstrates that HDL has a negative association with the risk of DPN, while FBG, TG, LDL, BMI, age, and the XRCC1 Arg399Gln SNP show positive associations. Although the XRCC1 Arg194Trp CT and CC genotypes exhibited high overall importance in the XGBoost model (Fig. 5), their near-zero SHAP values in Fig. 6 suggest that their influence on DPN risk varies across individuals. This contrast emphasizes that global feature importance and SHAP analysis provide complementary perspectives: the former reflects the general contribution of each variable to model performance, while the latter captures how these effects differ at the individual level. Figure 7 highlighted the significant correlations between the XRCC1 Arg194Trp SNP (notably the C/T genotype) and the XRCC1 Arg399Gln SNP (A/A genotype), as well as disease duration, age, and lipid profiles, with severe DPN. The findings indicate that genetic susceptibility, metabolic dysregulation, and clinical variables significantly affect disease complications.

## Discussion

Numerous studies have highlighted the significant role of various genetic loci in predisposing individuals to DPN^40–42^. Therefore, ongoing identification and refinement of genetic factors affecting disease susceptibility are essential. Previous studies suggest that increased OS, along with subsequent DNA damage and impaired repair mechanisms, significantly contributes to the pathogenesis, progression, and severity of diabetes mellitus (DM) and its associated complications [43 − 42]. This study aimed to investigate the association of XRCC1 DNA repair gene polymorphisms Arg399Gln (rs25487) and Arg194Trp (rs1799782) SNPS with the risk of T2DM and DPN in the Egyptian population while also examining correlations with clinical parameters and disease severity. This study is the first in Egypt to investigate XRCC1 polymorphisms in T2DM and DPN, utilizing machine learning algorithms to enhance predictive modeling and risk stratification.

Our findings indicate a significant association between the XRCC1 Arg399Gln SNP (rs25487) and an elevated risk of DPN. However, no association was found regarding the risk of developing T2DM. Further analysis revealed that the A allele elevated the risk of DPN approximately threefold when comparing the complicated group with the control group and twofold when compared to the uncomplicated T2DM group. These findings suggest that the XRCC1 Arg399Gln SNP may contribute to DPN pathogenesis rather than T2DM susceptibility. Our results align with prior research. For instance, Kasznicki et al.^46^ and Al-Musawi et al.^47^ observed no association between XRCC1 Arg399Gln SNP and T2DM susceptibility in a Polish and Iraqi populations, respectively. Consistent with our findings, Merecz et al.^48^ documented an elevated frequency of the XRCC1 Arg399Gln SNP in DPN patients, correlating with a 1.85-fold increased risk relative to controls. However, Yesil-Devecioglu et al.^26^ reported a significant association with T2DM (OR 3.09), contrasting our findings. In addition to DPN, various studies have associated XRCC1 Arg399Gln SNP with other complications of diabetes, such as diabetic retinopathy and nephropathy^26,49^. The discrepancy in T2DM association may arise from genetic or environmental differences among populations.

Regarding the XRCC1 Arg194Trp SNP (rs1799782), while this variant has been implicated in several diseases^50–53^, its direct association with T2DM and DPN remains understudied. While Merecz et al.^48^ reported no significant association, our research identified the XRCC1 Arg194Trp variant as a risk factor for T2DM and DPN, showing risk increases of up to twofold in most comparisons. The findings suggest that XRCC1 Arg194Trp is a genetic risk factor for DPN and T2DM specific to certain populations (Table 8).

Furthermore, haplotype analysis has been explored in prior research, with studies such as Przybylowska-Sygut et al.^54^ identifying an association between the 194Trp and 399Gln variants and heightened breast cancer risk. Conversely, Salimi et al.^55^ reported no correlation with systemic lupus erythematosus. This study is the first to investigate the association of their haplotypes with the risk of DPN and T2DM. We identified the ‘A-T’ haplotype as significantly associated with an increased risk of DPN, whereas the ‘A-C’ and ‘GT’ haplotypes were correlated with a higher risk of T2DM in patients without DPN. In contrast, the ‘G-T’ and ‘A-C’ haplotypes were linked to a reduced risk of DPN. The findings underscore the utility of XRCC1 haplotype analysis in identifying individuals at high risk, facilitating personalized risk assessments, and facilitating early interventions. A clarification for our results could be that although each allele independently contributes to the increased risk of DPN, the combinations of alleles (a haplotype) on the same chromosome create different effects based on a phenomenon named non-additive effect, in which the above-mentioned combinations significantly mask the risk effect of individual alleles or offer a protective effect as observed in our results^56,57^.

Concerning XRCC1 Arg399Gln, this polymorphism is found in exon 10 in the conserved BRCA1 carboxyl-terminal domain (BRCT1 domain and/or PARP’s central domain) of the gene, which includes 301–402 codons; thus, codon 399 is situated within this binding region. The XRCC1 gene serves as a common target for poly(ADP-ribosyl)ation in the BER and single-strand break repair (SSBR) pathways. Poly(ADP-ribose) polymerase 1 (PARP1) detects DNA damage, binds to the BRCT1 domain of XRCC1, and activates its function as a scaffold for assembling BER/SSBR machinery^58^. Prior reports demonstrated that the Arg399Gln polymorphism arises from a G-to-A substitution resulting in a non-conservative amino acid change (arginine to glutamine at codon 399), and the wild type (G/G) genotype is associated with normal gene activity, while the polymorphic (A/A) genotype alters protein repair activity by decreasing the capacity to remove oxidized DNA damage by 3–4 times and this alteration is connected to DNA repair efficiency and contributes to the risk of several diseases^59,60^.

In terms of XRCC1 Arg194Gln SNP results from a C-to-T substitution at codon 194 (position 26,304), leading to an Arg-to-Trp amino acid change^27,61^. In the wild-type genotype, XRCC1 interacts with DNA ligase III, DNA polymerase β (polyβ), and poly (ADP-ribose) polymerase (PARP) to form repair complexes. However, this polymorphism alters XRCC1’s structure and function, compromising DNA repair efficiency. Located near phosphothreonine-198 and within the linker region between the NH2-terminal and BRCT1 domains (positions 315–403), it may disrupt XRCC1’s interaction with proliferating cell nuclear antigen (PCNA), further affecting genomic stability^62^.

Regarding DPN severity, our study investigated the genotypic distribution of the aforementioned SNPs in relation to the TCNS and NDS systems or their association with DPN progression. We found that mild DPN complications correlated with wild-type genotypes, whereas severe DPN was predominantly associated mutant genotypes of both SNPs. These results suggest that the XRCC1 SNPs, when combined with DPN severity scores, could predict individuals at high risk of developing severe DPN. However, prior studies were in contrast with our outcomes^47,63^.

These findings prompted a comparison of TCNS and NDS efficacy in classifying DPN severity (mild, moderate, severe) using the XGBoost algorithm. TCNS demonstrated superior predictive performance. Its enhanced sensitivity facilitates early detection, while its specificity minimizes misclassification, underscoring its clinical utility for timely diagnosis and targeted treatment. These results align with prior studies^64,65^. Conversely, Nogueira et al.^66^ reported that NDS exhibited excellent sensitivity and specificity, correlating strongly with neuropathy severity.

Furthermore, we conducted traditional logistic regression analysis to identify risk factors influencing DPN. Our findings indicated that age, BMI, FBG, LDL, TG, 399G/A, and 399 A/A genotypes, along with the 194 C/T and 194T/T genotypes, were significantly correlated with an elevated risk of DPN. Consistent with our findings, numerous previous studies have demonstrated the relationship between age and the onset of DPN^67–69^. Callaghan et al.^70^ demonstrated that a higher BMI correlates with an elevated risk of DPN, as obesity is a recognized risk factor for complications associated with diabetes. Furthermore, the prevalence of neuropathy is significantly greater among obese individuals, including those without diabetes. Alshammari et al.^5^ reported that participants with diabetic neuropathy exhibited elevated BMI, FBG levels, cholesterol, LDL, and TG compared to those without neuropathy. Recent studies have corroborated our findings^61,71–73^. Nonetheless, numerous studies have contradicted our results^74–76^.

Machine learning (ML) models were utilized to identify risk factors and assess their interactions collectively, yielding greater accuracy than logistic regression while maintaining all variable information and providing a comprehensive interpretation of the most influential predictors of DPN risk. This aligns with Ravì et al.^77^ who highlighted the growing use of ML in medicine for predicting disease progression and offering significant insights. Similarly, Kavakiotis et al.^78^ emphasized the effectiveness of ML in diabetes prediction to mitigate disease progression and complications.

Our analysis showed that both RF and XGBoost attained high accuracy, reaching up to 98%, in the detection of complications. The SHAP method revealed that LDL, FBG, HDL, TG, BMI, and the XRCC1 399 A/A genotype are the primary factors influencing complication risk. The findings are consistent with those of Shiren et al.^79^ who established the efficacy of RF in predicting diabetic complications (AUC = 82%) and highlighted the significance of monitoring key biomarkers. Similarly, Wu et al.^80^ and Lian et al.^81^ reported that XGBoost demonstrates strong predictive power in DPN, achieving accuracies of 85.4% and 74.6%, respectively. The well-balanced cohort in our study, comprising 256 controls, 247 individuals with uncomplicated diabetes, and 229 with diabetic complications, likely enhanced the model’s performance. Conversely, Shin et al.^82^ examined DSPN prediction utilizing a limited and imbalanced dataset, specifically within the “probable” group (*n* = 31), resulting in reduced machine learning accuracy (≤ 74%). This emphasizes the benefit of our study’s comprehensive dataset in increasing predictive accuracy and improving the management of diabetic complications, highlighting the necessity of well-structured data to develop reliable models and draw significant conclusions.

## Conclusions

The association between the examined SNPs and DPN risk suggests that these genetic variants may play a role in individual susceptibility to DPN. Identifying individuals with genetic predispositions, such as carriers of the XRCC1 Arg399Gln and Arg194Trp polymorphic variants, and subjecting them to regular follow-ups may aid in disease prevention or facilitate early diagnosis. Moreover, comprehending the genetic underpinnings of DPN offers significant insights for prognosis and personalized medicine, enabling early interventions for individuals at high risk. Moreover, machine learning models are essential for evaluating the risk of DPN by synthesizing genetic and clinical data, enhancing predictive accuracy, and informing targeted prevention strategies.

### Recommendations

Further studies are needed to elucidate the intricate relationship between genetic and environmental influences on disease severity. Furthermore, it is crucial to examine additional SNPs in the XRCC1 gene such as Arg280His (rs25489) SNP and their interaction with the aforementioned SNPs to identify more reliable predictive genetic factors. Additionally, the analysis of antioxidant enzyme activities such as catalase (CAT), superoxide dismutase (SOD), glutathione peroxidase (GPX) and total antioxidant status (TAS) and their relationship with DPN should be incorporated into future research.

## Supplementary Information

Below is the link to the electronic supplementary material.

Supplementary Material 1

## Data Availability

The datasets produced and/or analyzed throughout this current investigation are not available to the public but can be taken from the corresponding author upon a reasonable request.

## Abbreviations

ML: Machine Learning
RF: Random Forest
XGboost: Extreme Gradient boosting
OR: Odds ratio
HWE: Hardy-Weinberg equilibrium
AIC: Akaike information criteria
