# Supplementary material for: Genetic polymorphisms in DNA repair gene XRCC1 and the risk of diabetic polyneuropathy
Source: Sci Rep. 2026 Feb 3;16:4815. doi: 10.1038/s41598-026-35213-1 (PMC12873327; doi:10.1038/s41598-026-35213-1)
Supplement: Supplementary file 1 — Supplementary Material 1 [file 41598_2026_35213_MOESM1_ESM.docx]

**Table: XGBoost hyperparameter settings**

| hyperparameters | values |
| --- | --- |
| max_depth | 15 |
| min_child_weight | 12 |
| colsample_bytree | 0 |
| subsample | 1 |
| gamma | 0.8 |
| Max_delta_step | 5 |
| booster | “gbtree” |
